# 1165. Ascertainment of anthropometric thresholds to identify glucose intolerance in HIV-endemic African populations: results from a population-based survey in rural KwaZulu-Natal, South Africa

**DOI:** 10.1093/ofid/ofac492.1002

**Published:** 2022-12-15

**Authors:** Alison Castle, Mark Siedner

**Affiliations:** Massachusetts General Hospital, Boston, Massachusetts; Harvard Medical School, Boston, Massachusetts

## Abstract

**Background:**

Improved screening and diagnostic algorithms are needed to achieve 2030 targets proposed by the Global Diabetes Compact. We sought to explore anthropometric thresholds to optimally screen and refer individuals for glucose intolerance testing in an HIV-endemic rural South African population.

**Methods:**

We analyzed data from the cross-sectional Vukuzazi study that enrolled adults living in rural KwaZulu-Natal, South Africa. We assessed screening thresholds for waist circumference (WC), body mass index (BMI), and waist hip ratio (WHR) to detect glucose intolerance (hemoglobin A1c >6.5%) using weighted, non-parametric ROC regression analyses. We then assessed the diagnostic validity of traditional obesity thresholds, explored optimal thresholds for the South African population, and fit models stratified by sex, age, and HIV.

**Results:**

In the total cohort (n=17,868), the prevalence of glucose intolerance was 7.8% (95% CI 7.4–8.2) and HIV prevalence was 33.8% (95% CI 33.1-34.5). WC outperformed BMI and WHR in detecting glucose intolerance (males p< 0.001; females p< 0.001). Whereas guideline WC thresholds for females performed well for glucose intolerance (Sensitivity 91% [95% CI 89.5-92.5], positive predictive value [PPV] 14.9%), substantially lower thresholds were needed to achieve acceptable sensitivity and positive predictive values among men. We found significant sub-groups effects by sex and age, but HIV status did not affect WC thresholds for identifying glucose intolerance.

**Conclusion:**

Waist circumference improves anthropometric screening for glucose intolerance in South Africa. Guideline-based WC thresholds are appropriate for females, but male-derived WC cutoffs were optimized at lower thresholds. HIV did not affect the performance of WC thresholds in either sex. In rural African populations, thresholds that maximize specificity and PPV for efficient resource allocation may be preferred.

**Disclosures:**

**All Authors**: No reported disclosures.

